# Long-Term Sea Surface Variability Regarding Seafloor Topography

**DOI:** 10.3390/s25206391

**Published:** 2025-10-16

**Authors:** Magdalena Idzikowska, Katarzyna Pajak, Kamil Kowalczyk

**Affiliations:** Department of Geoinformation and Cartography, Institute of Geodesy and Civil Engineering, University of Warmia and Mazury in Olsztyn, Oczapowskiego St. 2, 10-719 Olsztyn, Poland; katarzyna.pajak@uwm.edu.pl (K.P.); kamil.kowalczyk@uwm.edu.pl (K.K.)

**Keywords:** radar altimetry, bathymetry, sea level anomaly, seafloor topography, free-air gravity anomaly, mean dynamic ocean topography

## Abstract

This study presents an analysis of regional sea level variability on various seafloor structures. The main aim of this paper was to determine the regional trends of sea level changes (time span of 29 years) in the area of the ocean trench and submarine canyon, in the area of seamounts and corrugations, and then to compare the models of the seafloor with the models of the sea surface. We used hybrid datasets, including satellite altimetric time series, multibeam bathymetric soundings, GEBCO products, free-air gravity anomaly models, and mean dynamic ocean topography models. Radar remote sensing and spaceborne radar technologies are essential in capturing the long-term dynamics of sea surface variability regarding seafloor topography. The values of regional sea level change trends in the seamounts and corrugation region are two times higher (from 2.56 ± 0.10 mm/year to 7.66 ± 0.18 mm/year) than in the trench and canyon region (1.75 ± 0.01 mm/year to 3.65 ± 0.07 mm/year). In the region of trench and canyon, i.e., on narrow and deep forms of the seafloor, the values of regional trends are stable and regular. In the region of seamounts and corrugations, where the depth is more diverse, regional trend values are higher and irregular. Study results show that regional sea level fluctuations can be the consequence of the diversified structure of the seafloor. The region of the trench and canyon, although characterized by high susceptibility to climatic phenomena, presents lower amplitudes of sea level changes than the subregion of seamounts and corrugations, where the bathymetry of the seafloor is more complex.

## 1. Introduction

The global mean sea level (GMSL) is constantly increasing: in some regions, sea level rise (SLR) is three times the global average value (more than 3.00 mm/year) [1,2]. However, significant regional variations are observed: while in some locations, sea levels are rising more rapidly and higher than the global average value, in others, the rise is slower or even negative [3]. Global trends primarily reflect the overall reaction of the climate to the warming of the atmosphere and oceans, while regional variability is the result of tectonic and oceanographic processes and local bathymetric relief. Understanding global sea level trends and the processes responsible for regional differences in sea level is crucial for forecasting future changes and developing effective adaptation strategies to SLR [4].

The complex dynamics of sea level result from the interaction of many physical factors, and its analysis requires the integration of oceanographic, atmospheric, and geophysical data to understand processes on a local and global scale. The interaction between the sea and the atmosphere leads to complex dynamic phenomena such as wind waves, storm waves, tides, sea level fluctuations, and ocean currents. They directly affect the transfer of water masses in the oceans as well as global atmospheric circulation [5]. Wind interacting with the water surface generates waves and ocean currents, which are modulated by the Coriolis effect. According to Ekman’s spiral theory, the direction of flow changes with depth, leading to the concentration of water masses in major ocean currents [6]. In addition, the gravitational forces of the Sun and Moon cause periodic rises and falls in water levels, which generate tides, modifying local hydrostatic pressure and affecting the distribution of currents [7]. Atmospheric pressure causes local uplifts or subsidence of the ocean surface, detected, among others, by altimetry satellites [8]. Gravity from large topographic features, such as islands or undersea mountains, also pulls water toward them, leading to local disturbances in sea level and current distribution [9]. In addition, differences in temperature and salinity affect the density of seawater, causing thermohaline circulation. This process drives the global deep ocean current system responsible for transporting heat and salt between oceans [10,11]. Assessing sea level fluctuations remains challenging due to the influence of a wide range of climatic phenomena, such as El Niño and La Niña, PDO (Pacific Decadal Oscillation), NAO (North Atlantic Oscillation), and IOD (Indian Ocean Dipole), which can accelerate or slow down the rate of sea level rise [12,13,14,15]. It is estimated that global sea levels have risen by more than 20 cm over the past 140 years, highlighting the importance of monitoring and reporting sea level variability in the context of sustainable development research, particularly in vulnerable coastal areas [16,17,18].

Free-air gravity anomalies associated with the topography of the ocean floor are significant in local differences in sea level. Radar altimeters measure the sea surface height while at the same time detecting the gravity field on this surface [19]. For more than 30 years, a high correlation has been presented between gravity anomalies and depth—the available literature reports that about 1 mGal of gravity anomaly equals 1μrad of sea surface gradient [20]. These anomalies result, among other things, from the process of isostatic compensation, which describes the balance between the Earth’s crust and mantle. Under conditions of a homogeneous seafloor structure and a thin layer of sediments, changes in bathymetry remain linearly correlated with gravitational anomalies. Anomalies in free air are particularly important, as they accurately reflect uncompensated ocean floor structures, such as young ocean ridges [21]. Short wavelengths remain undetectable, while longer ones are largely suppressed by isostatic compensation. As a result, the topography of the seafloor is reflected in the gravitational field and in the shape of the ocean surface. The GOCE and GRACE satellite missions provide high-quality global data on the gravitational field [22,23,24], but due to their orbital altitude, they do not record signals associated with small bathymetric structures. Contemporary research indicates that integrating gravitational anomalies with altimetric measurements of sea surface height allows for significant improvements in bathymetric models, particularly in areas without direct sonar measurements [25,26,27,28]. The analysis of gravity anomalies in the context of isostatic compensation is a significant tool for reconstructing the topography of the seafloor and is widely used in geological and tectonic research and in the search for natural resources.

Previous studies have been focused primarily on integrating and filtering bathymetric and gravity data to improve model accuracy, as well as on improving bathymetric modeling using altimetry data [29,30]. However, to date, no analysis of sea level changes in relation to ocean floor topography has been conducted. This study addresses this gap by proposing an approach that combines hybrid datasets—satellite altimetry time series, free-air gravity anomaly models, multibeam echosounding data, the GEBCO model, and mean dynamic topography (MDT) models.

This study aims to determine the seasonal sea level variability and compare numerical models of depth, free-air gravity anomalies, and MDT (mean dynamic topography) to assess the relation between the seafloor relief and sea level changes. The research is based on hybrid datasets on diversified seafloor forms (submarine canyon, ocean trench, seamounts, and corrugations). An original feature of this approach is the direct connection between sea level changes and seafloor topography, which is an important addition to previous studies that focused mainly on precise bathymetric modeling.

An analysis of sea level variability in the context of seafloor topography offers valuable insights for the maritime economy, particularly in the preparation of environmental expert reports and impact assessments, which are an important part of investment planning related to the maritime economy. The results contribute to the broader context of climate change mitigation by opening new avenues for forecasting and evaluating risks in coastal locations. Understanding how sea level changes respond to the seafloor structures can also inform coastal development policies, such as the formation of building regulations and the establishment of safe setback distances from the shoreline. In addition, the results may support sustainable fisheries management and the designation of marine protected areas, thereby promoting sustainable stewardship of the marine environment.

The structure of this paper is as follows: Section 2 outlines the study area, with particular attention to its geographical and environmental characteristics. Section 3 introduces the materials used, and Section 4 provides a detailed account of the adopted methodology, including analytical procedures and applied models. Section 5 presents the research findings, which are interpreted in Section 6 in the context of previous studies and theoretical frameworks. Finally, Section 7 offers concluding remarks along with perspectives for future research.

## 2. Study Area

The study area is divided into two subregions with distinctly varied seafloor topography, allowing for a comprehensive analysis of how different seafloor forms affect sea level variability. This makes it possible to better understand how structures such as ocean trenches, submarine canyons, seamounts, and corrugations regulate local sea level trends. Considering the long-term and interdisciplinary scope of the analysis, the study area corresponds to the regions analyzed in prior studies [31].

The first subregion is the part of the west coast of the South America–Peru–Chile Trench and San Antonio submarine canyon (32° S < φ < 33.5° S, 72° W < λ < 73° W). This is the longest trench in the Pacific Ocean, stretching 5900 km from Ecuador to Chile, with an area of about 590,000 km^2^. The depth exceeds 8000 m. Due to specific geological, geotectonic, geomorphic, and climatic conditions, the Atacama Trench region is vulnerable to frequent earthquakes, local tsunamis, volcanism, and geological hazards such as floods, landslides, and mass movements. This is due to its location in the subduction zone, where the Nazca oceanic plate slides under the South American continental plate [32]. Based on the GEBCO model, depths in this region range from −6176.0 m to −843.0 m. Multibeam bathymetric soundings (NOAA NCEI resources) were arranged irregularly, so integration with the GEBCO model was necessary. In the first subregion, 31 virtual observation points were determined (points T1–T15 (trench), C1–C10 (canyon), and A1–A6 (additional)).

**Figure 1:**
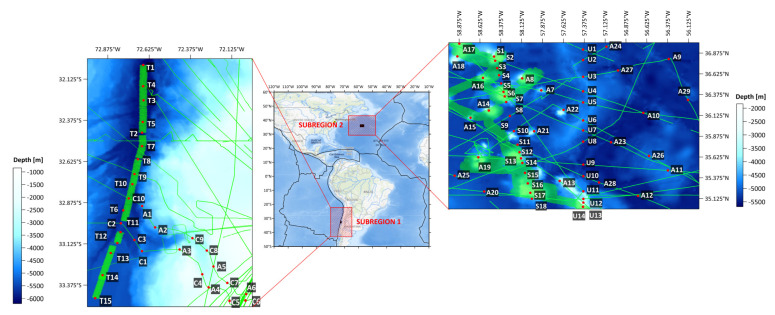
Study area. Multibeam survey tracklines (green lines), virtual observation points (red dots), and tectonic plate boundaries (black lines).

The second subregion is a seamount chain and the area of corrugations (35° N < φ < 37° N, 56° W < λ < 59° W) known as the New England Seamounts and Corner Rise Seamount Complex. The volcanic activity that formed this long chain has been the history of North American and African plate movements for more than 100 million years. The seamount chains (extinct volcanoes) New England Seamounts and Corner Rise Seamount Complex extend from the Mid-Atlantic Ridge (MAR) to the east coast of the United States of America. The second subregion is a biodiverse environment and an interesting ocean circulation research area. Seamounts regulate the movement of seawater, change the direction of ocean currents, and affect the distribution of temperature and salt in ocean basins. This allows for an analysis of the interaction between seafloor topography and the sea surface. Based on the GEBCO model, the maximum depths in this region are −5683.00 m, while the minimum depths oscillate to −1838.00 m. The seabed of this region was partially mapped in 2021 by NOAA Ocean Exploration and its partners during the “2021 North Atlantic Stepping Stones: New England and Corner Rise Seamounts” expedition. This expedition used remotely operated vehicles (ROVs) at depths exceeding 4000 m to collect baseline information on unknown deep-sea objects off the US East Coast. These data were used in the study to better understand the seafloor diversity and distribution of deep-sea habitats, which is important in decisions about managing marine resources [33]. In the second subregion, 55 virtual observation points were determined (points S1–S18 (seamounts), U1–U14 (corrugations), and A7–A29 (additional)). The study area is presented in Figure 1.

## 3. Materials

### 3.1. Satellite Altimetry Data

This study was carried out using European Copernicus Marine Service—part of the EU’s Space Programme, focused on Earth observation and dedicated to monitoring our planet and its environment for the benefit of all European citizens. CMEMS (Copernicus Marine Environment Monitoring Service) provides free, systematic, and reliable data on the state of the blue (physical), white (sea ice), and green (biogeochemical) oceans on a global and regional scale [34]. The originating centre of the dataset is CLS (Collecte Localisation Satellites), located in Ramonville-Saint-Agne (France). CMEMS data are essential in the monitoring of sea level variations, in the analysis of ocean currents, storm waves, and tides, and in the calibration of ocean and climate circulation. Altimetry products—such as sea surface height and ocean current data enable integration with complementary satellite observations, including thermal, gravity, and salinity data, thereby supporting comprehensive analyses of physical and climatic processes on regional and global scales [35]. In oceanographic research, these data are used to assess sea-level variability, model ocean circulation, and monitor biogeochemical processes. Their integration with other information sources enables comprehensive studies of the state and dynamics of the oceans, which is essential for effective marine environment management and for planning climate change adaptation strategies.

In this study, time series of sea surface heights above sea level (daily, gridded sea level anomalies) for each virtual observation point, a time span from 1 January 1993 to 31 December 2021, with a spatial resolution of 0.25° × 0.25° were acquired (product id: SEALEVEL_GLO_PHY_L4_MY_008_047) [36]. The available sea level anomalies are a multi-year (MY) level-4 gridded product (L4), which is calculated as the difference between the instantaneous sea surface height (SSH) and the time reference. Mean sea surface (MSS) can be the reference—in the case of the selected product, the mean MSS is from 1993 to 2012. Sea level anomalies are gridded products derived through optimal interpolation, combining observations from multiple available altimetry missions. This product is processed by the DUACS (Data Unification and Altimeter Combination System) multimission altimeter data processing system [37].

Processing details are available at http://duacs.cls.fr (accessed on 20 May 2024). The datasets are expanded four times a year and cover the period from 1993 to 5–9 months before the present. The available datasets use the highest quality altimetry measurements and geophysical corrections and are compiled using a unique system to minimize the risk of quality loss or the appearance of false signals over time. The Multi-Year Interim series (MY_INT) covers the latest observations in recent months, using new products, including actual standards and altimeter corrections. This dataset was corrected for atmospheric effects (ionospheric delay and dry/wet tropospheric effects) and geophysical processes (solid, ocean, pole tides, loading effect of ocean tides, sea state bias, and the inverted barometer response of the ocean). Detailed information about data precision and corrections can be found on the AVISO (https://www.aviso.altimetry.fr/en/home.html—accessed on 20 May 2024) and CMEMS (https://marine.copernicus.eu/pl—accessed on 20 May 2024) websites. In addition, a User Manual and a Quality Information Document are provided for every dataset on the industry pages. Global and regional sea level deviations that may be introduced by changes in standards/corrections are managed by DUACS processing to ensure sea level continuity and a simple transition for users. The complete reprocessing of the multi-year series is usually delivered every three or four years. In this study, the CMEMS product (OMI_CLIMATE_SL_GLOBAL_regional_trends) [38] contains a tendency of sea surface height above sea level (SSH) in the time span from 1 January 1993, and a spatial resolution of 0.25 deg × 0.25 deg was used in the validation process. It is a multi-year, gridded model. At each grid point, the trends and accelerations are estimated on the time series corrected from global TOPEX-A instrumental drift [39] and GIA correction [40] and adjusted from annual and semi-annual signals. Regional uncertainties on the trends and processing data information can be found in [41] and in the Quality Information Document attached for each CMEMS product (https://data.marine.copernicus.eu/products, accessed on 20 May 2024).

### 3.2. Multibeam Bathymetry Data

The National Centers for Environmental Information (NCEI) of NOAA (Maryland, USA) in collaboration with the International Hydrographic Organization (IHO) Data Center for Digital Bathymetry (DCDB) in Monaco, archives and distributes bathymetric data collected by hydrographic, oceanographic, and industrial vessels and platforms during surveys and cruises. These data are used in many national and international bathymetric mapping projects and are free of charge for users without any limitations. The multibeam bathymetric data were obtained from IHO Data Centre for Digital Bathymetry (DCDB) collections (https://www.ncei.noaa.gov/iho-data-centre-digital-bathymetry—accessed on 15 February 2023) and include 23 irregular multibeam bathymetric survey tracklines (time span 1988–2017 in subregion 1) and 17 tracklines (time span 1984–2021 in subregion 2). Raw, sonar data files were decompiled and converted to a usable format (.txt) using MB-System software (5.7.8 version). 

### 3.3. Digital GEBCO Model

The study has applied the General Bathymetric Chart of the Oceans (GEBCO_22) model, which is publicly available and hosted by the British Oceanographic Data Centre (BODC) in Liverpool (United Kingdom). The GEBCO_22 model is a global, digital elevation model (DEM) for ocean and land with a spatial sampling interval of 15 arc sec (elevation in meters, at the center of grid cells). GEBCO products are a compilation of bathymetric data from different sources of various quality and range. Lemenkova [42] emphasized that due to regular updates to the GEBCO grid, the inclusion of the latest soundings in the datasets, and hydrographic meetings, the 15 arc sec resolution datasets are a reliable source for ocean floor modeling and mapping. The base of the GEBCO_22 model is the SRTM15+ V2.4 dataset (comparable with version 2.1) [43]. The SRTM15+V2.4 data were completed with 905 new, multibeam sonar soundings and grids from INFREMER resources (https://www.ifremer.fr/fr—accessed on 6 March 2023) by the Regional Centers of the Seabed 2030 project, which were provided to the Global Center to create the GEBCO_2022 grid. The GEBCO_22 model was produced using the ‘remove–restore’ method. This is the two-step process of calculating the difference between the new data and the ‘base’ grid, then meshing the difference and adding it back to the existing “base” grid. The goal is to achieve a seamless transition between ‘new’ and ‘baseline’ datasets with minimal disturbance to the existing baseline dataset [44,45]. Predicted depths obtained from satellite altimetry in the GEBCO_22 model are based on the V31 gravity model [46]. 

### 3.4. SIO Model Gravity

The first preparation for mapping the marine gravity field was connected with the launch of the GEOSAT geodetic mission in 1985 [46]. In this study, the latest version of the free-air gravity anomaly model—SIO V32—was obtained from the repository of the Scripps Institution of Oceanography at the University of California in San Diego (USA). This model is a gridded product with 1 min (≈0.0167 deg) spacing, available in netCDF format since 1997 (starting with version 7.2). Since then, models have been regularly improved using data from new satellite missions or new processing methods [47,48]. Products are available on the industry site (https://topex.ucsd.edu/pub/global_grav_1min/, accessed on 19 July 2024). 

### 3.5. DTU 2022 Mean Dynamic Topography Model

Mean dynamic ocean topography (MDT) represents the differences between the time-averaged sea surface and the geoid, thereby reflecting the mean oceanic circulation. The geodetic mean dynamic ocean topography model DTU22MDT (distributed by the Technical University of Denmark in Kongens Lyngby) was derived using the DTU21MSS mean sea surface model, which has been estimated based on multimission altimetric data (from 1993 to 2012). For MDT model generation, the geoid model XGM2019e was used, which is based on marine gravity from satellite altimetry. General derivation of the geodetic MDT relies on a processing scheme that applies spatial filtering to suppress errors. The model is available in GRAVSOFT grid format (in ASCII) or XYZ ASCII file format with 0.125° × 0.125° spatial resolution. Further information regarding data quality and processing can be found in the referenced sources [49,50,51].

## 4. Methodology

This study focused on estimating seasonal variations in sea level and comparing the results with seafloor structure. The analysis was based on daily time series of sea level anomalies (SLAs) from 1993 to 2021, obtained from the CMEMS database. Preliminary data processing included quality control, outlier removal, and interpolation of missing values to ensure signal continuity and consistency. Linear regression using the least squares method was applied to remove the long-term trend. Detrending improved the identification of periodic components and reduced signal non-stationarity [52].

In harmonic analysis, a functional model is built that is the sum of harmonics—sine and cosine functions over a specified time interval [53,54,55]. Harmonic values are estimated according to the length of the time series. In the model, it is sufficient to include only the most relevant harmonics rather than all possible components. Annual and semi-annual harmonics were calculated (the amplitudes and phase shift were calculated for each harmonic). In addition, an 18.61-year harmonic was estimated to examine whether the time series exhibits fluctuations associated with the 18.6-year lunar nodal cycle. The 18.61-year is a lunar nodal cycle caused by the Moon’s relative motions. This important precession of the Moon causes tidal modulations on some interannual time scales. These modulations affect the interpretation of data spanning several years, especially for extreme water levels [56,57]. The 18.61-year nodal cycle of the Moon has a direct impact on sea levels and tidal ranges at the coast, which requires a detailed analysis of long-term sea level observations [57,58]. The harmonic function is used for time series analysis and is based on a transition from the time domain to the frequency domain, which decomposes the input signal into a series of periodic functions, such that the individual frequencies make up the original function [59]. The time series is disturbed by periodicity components, so the harmonic structure of the series was analyzed, and using the harmonic values, the long-term trends of sea level change were redetermined according to the following formula [60]:

(1) $$f_{h}\left(t\right)=a+bt+\sum_{i=1}^{N}\left(A_{i}sin\left(\omega_{i}t\right)+B_{i}cos\left(\omega_{i}t\right)\right)+\epsilon (t)$$

where $f_{h}\left(t\right)$ is a harmonic function; a means bias; b means linear trend; t means time; $A_{i}\;and\;B_{i}$ are the amplitudes of the periodic components of frequency $\omega_{i}$; *N* is the number of periodic components; and ϵ is the residual SLA. The units are millimeters/year.

Harmonics with shorter or longer periods were excluded due to their limited statistical and physical significance in the context of the period under study and the characteristics of the data (lack of clear signals in the spectrum [61]. The selection of harmonics considers components with periods corresponding to annual, semi-annual, and the 18.61-year lunar cycle, which have physical foundations and a significant impact on sea level. Annual and semi-annual harmonics were selected because of their predominant influence on seasonal sea level variability, which is closely linked to climate seasonality, solar radiation, and cyclical atmospheric–oceanic processes. The 18.61-year harmonic corresponds to the well-established lunar nodal cycle, which modulates tidal patterns over long time scales. Incorporating this component enables the assessment of whether such lunar–tidal effects are detectable in the analyzed data, which may be important for interpreting long-term trends and changes in extreme sea levels [62,63,64].

To validate the regional sea level trends obtained from harmonic analysis, wavelet transform (WT) was also employed, enabling decomposition of the signal into time–frequency components. This approach facilitates the identification of temporal variability in cycle amplitudes, which is especially valuable for analyzing signals with non-stationary characteristics [65].

Continuous wavelet transform (CWT) enables signal localization in both the time and frequency domains. CWT reflects the correlation between the analyzed continuous signal and a function called the analyzing wave or mother wave, which is defined by the following equation:

(2) $$W\left(a,b\right)=\frac{1}{\sqrt{a}}\int_{-\infty }^{\infty }f\left(t\right)\psi^{*}\left(\frac{t-b}{a}\right)dt$$

where $\psi^{*}\left(t\right)$ is the complex conjugate of the analyzing mother wavelet *ψ*(*t*); a is the scale of the wavelet; b is the translation parameter of the wavelet; $\frac{1}{\sqrt{a}}$ is the factor that represents the normalized energy (the energy of the wavelet must be the same for different a values of the scale).

The selection of the mother wavelet, or analyzing wavelet function, is application-dependent and is not governed by a standardized procedure. A commonly recommended criterion is to choose a wavelet function whose shape closely resembles the characteristics of the signal under investigation. In the present study, the Daubechies 4 (db4) wavelet was adopted as the mother wavelet [66,67,68].

The trend analysis was implemented using linear and quadratic regression methods. To assess the cyclicality of the signal, Fast Fourier Transform (FFT) was applied to identify the amplitudes of annual and semi-annual cycles. The procedure enabled a comprehensive assessment of sea level dynamics, and the results of the wavelet analysis served as an independent confirmation of the presence of significant cycles in the data.

The Kalman filter method was used to validate the estimated trends and seasonal components. The Kalman filter has enabled the determination of trends and amplitudes (at a few selected stations), which were compared with the results of harmonic analysis. Combining the Kalman filter, linear regression (OLS and robust), and harmonic regression to estimate trends and seasonality in (SLA) sea level anomaly time series. The Kalman filter iteratively updated the estimation based on the state equation and observations, reducing the measurement noise and smoothing the series [69,70] by the following equations:

(3) xk=Fxk−1+wk−1   F=1101

(4) y~k=Hxk+vk   H=10

where $x_{k}=\left[\begin{array}{c} {level}_{k}\\ {trend}_{k} \end{array}\right]$ is the state vector at time *k*; F is the state transition matrix; $w_{k-1}$ is the noise vector with normal distribution N(0,Q); $y_{k}$ is the observation in time; H is a measurement matrix; $v_{k}$ is the noise with normal distribution N(0,R); Q is the covariance of noise; R is the variance of noise.

**Figure 2:**
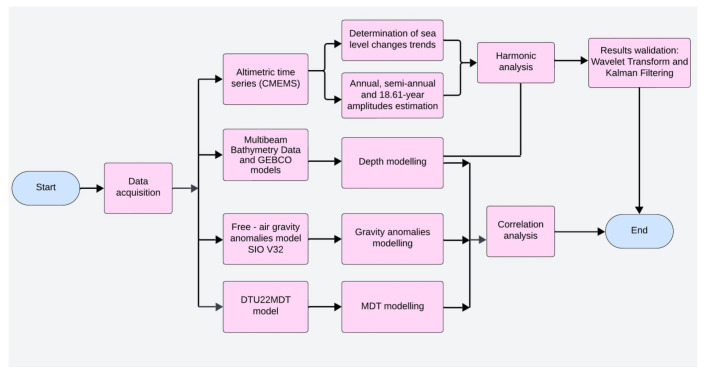
Flow chart of the processing steps.

The combined application of linear regression, harmonic analysis, wavelet transform, and Kalman filtering enabled comprehensive modeling and validation of both seasonal and long-term sea level variability. The selection of harmonics was guided by physical principles and spectral characteristics of the data, while the complementary use of filtering and time–frequency analysis enhanced the reliability and interpretability of the results. Multibeam echosounding data were converted to a usable format, extracting information on location, depth, and times, using the MB-System (https://www3.mbari.org/data/mbsystem/index_ldeo.html—accessed on 26 February 2023). This is an open-source software package for processing and displaying seafloor mapping data, particularly bathymetric data and backscatter images acquired from multibeam, interferometry, and sidescan sonars. The processed depths were integrated with GEBCO model values. The depth model (based on multibeam and GEBCO values), free-air gravity anomaly model (based on anomalies from the SIO V32 model), and MDT model were visualized. In the study, Pearson’s correlation coefficient was selected as the method for assessing spatial relationships between geophysical models (depth, free-air gravity anomalies, and mean dynamic topography)—correlograms in a 0.25° × 0.25° grid. Pearson’s correlation coefficient was selected due to its established use in geospatial and oceanographic studies for assessing relationships [71]. Furthermore, the visualization of Pearson’s correlation in the form of spatial correlograms facilitates the simple interpretation of regional relationships within a uniform grid (0.25° × 0.25°). Given the objectives of this analysis—the identification and visualization of spatial correlations in large geophysical datasets related to the marine environment—a flow chart of the study process is shown in Figure 2.

This study used an approach that integrated data with different spatial resolutions, formats, and time ranges, combining static and quasi-static models (GEBCO 2022, SIO V32, MDT22) with dynamic SLA altimetry data. Static data reflect seafloor topography and tectonic structures, while dynamic data record seasonal and long-term sea level changes. The diversity of the datasets required consideration of potential temporal inconsistencies, differences in resolution, and inherent instrumental uncertainties and errors resulting from altimetry and bathymetry processes. The quality control process included validation and standardization of all datasets prior to integration. SLA data were processed with geophysical corrections and outlier detection, the GEBCO_22 model was checked for coastal artifacts and combined with multibeam data, and MDT22 and SIO V32 were verified for spatial consistency and compliance with the WGS84 reference system. Data integration used Ordinary Kriging interpolation, and missing data were excluded from further analysis. SLA trend analysis included separation of temporal components using harmonic methods and CWT and Kalman filtering. Methodological limitations include the linear nature of correlation measures, limited spatial resolution, and potential time lags between datasets. Despite these limitations, the approach adopted allows for a reliable interpretation of spatial relationships between sea level dynamics and the geophysical structure of the ocean floor, setting directions for further research.

In summary, the methodology used in this study, despite its complexity, has several limitations. The study of the relationship between seafloor topography and sea level change was conducted using data with varying characteristics—static bathymetric and geophysical models (GEBCO, MDT, SIO V32) and dynamic altimetry data (SLA and CMEMS). Harmonic analysis was used to determine trends in order to reduce noise and separate time components. The limitations of the analysis include, among others, the linear nature of the correlation measure used, potential time shifts between datasets, and limited spatial resolution, which may affect the accuracy of the mapping of local processes. Nevertheless, the approach adopted provides a solid basis for further, more detailed analysis. The limitations indicated do not undermine the reliability of the results but rather point to directions for further research.

## 5. Results

**Figure 3:**
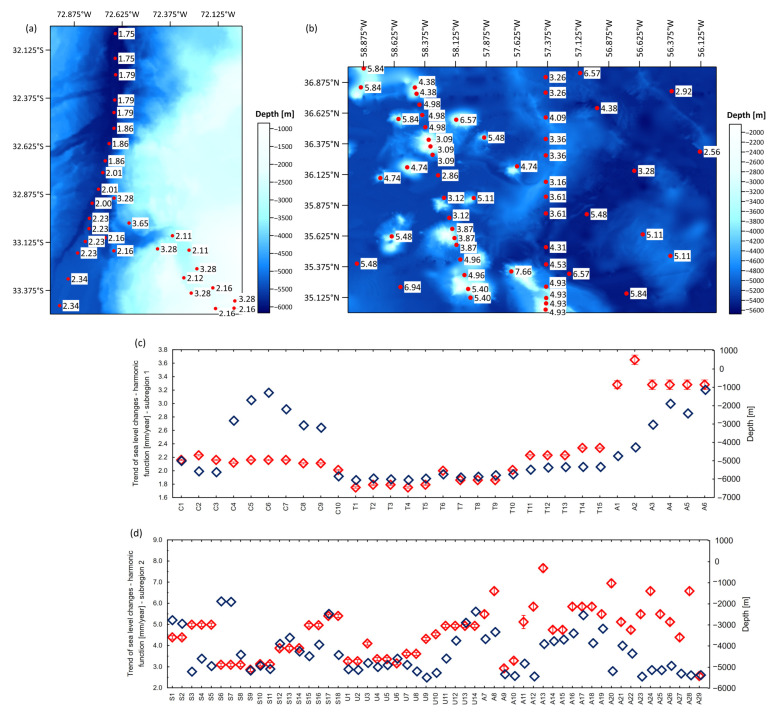
Trends of sea level changes determined by harmonic analysis—trench and canyon region (**a**), seamounts and corrugation region (**b**), and combination of trend changes relative to depth (**c**,**d**). The trend units are in mm/year. Regional sea level trends obtained through harmonic analysis are represented by red squares, while blue squares correspond to the depths.

Based on the acquired SLA time series at all virtual stations, trends in sea level changes using a harmonic function were determined. Trend values in both subregions are shown in Figure 3.

**Figure 4:**
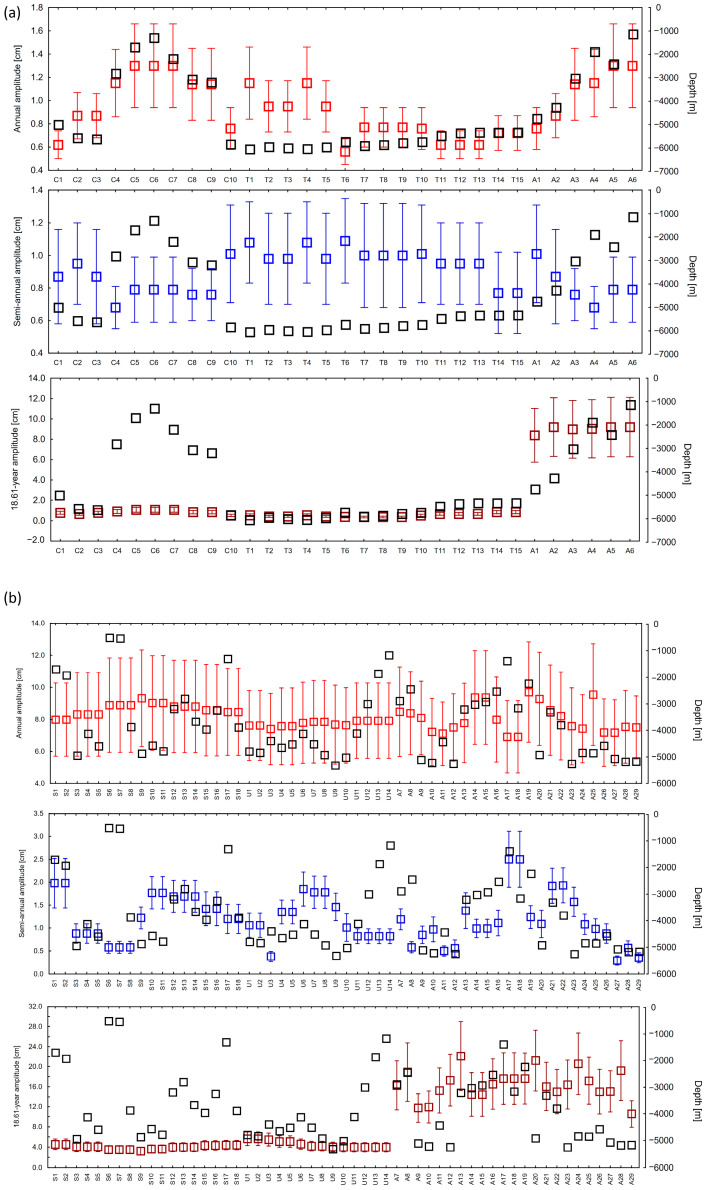
Amplitudes of the annual, semi-annual, and 18.61-year cycle—ocean trench and canyon region (**a**), region of seamounts, and undulating seafloor (**b**). Black squares represent depth in meters. The amplitudes are in centimeters.

The values of sea level change trends in the ocean trench and canyon region (subregion 1) are in the range of 1.75 ± 0.01 mm/year to 3.65 ± 0.07 mm/year. In depths > 5000 m, trend values are less than 2.00 mm/year. In the canyon area, where the depth decreases (<3000 m), the trend values increase and exceed 3.00 mm/year. In the area of seamounts and corrugations, the seafloor is more varied, and the trend values are higher (from 2.56 ± 0.10 mm/year to 7.66 ± 0.18 mm/year). Regarding the results, we can say that the more the structure of the seafloor is diverse, the higher the jumps are in the trend values of sea level changes. In the deep parts of the ocean basin, the trend values are considerably lower, and on the top of seamounts, they are several times higher. Altimetric time series are affected by seasonal signals, so the seasonal dynamic of the sea surface at selected locations was investigated using harmonic analysis. Annual, semi-annual cycles, and the 18.61-year lunar nodal cycle, which directly affects high tidal levels on a global scale, were determined. The results are shown in Figure 4.

In the trench and canyon area, annual and semi-annual amplitudes remain relatively low, not exceeding 2.0 cm. At points in deeper locations than 4000 m, annual amplitudes fall below 1.0 cm, and semi-annual amplitudes range from 0.7 to 1.1 cm, with errors of ~0.3 cm. The exceptions are points at a depth of about 6000 m, where semi-annual amplitudes exceed 1.0 cm. The amplitudes associated with the 18.61-year cycle are stable and low (0.5–1.1 cm) in this area, with errors of up to 0.2 cm. Changes in depth (e.g., in stations C4–C9) have not significantly affected these values. In contrast, at stations A1–A6, with increasing depth, a significant jump in 18.61-year amplitudes is observed, reaching values of 9–12 cm.

In the region of seamounts and corrugations, annual amplitudes are significantly higher, approximately 8.0–9.5 cm, with errors of 2.5–3.0 cm. Semi-annual amplitudes in these areas do not exceed 2.5 cm, and their errors are relatively low (~0.3 cm). Amplitudes in the 18.61-year cycle reach values of 3.5–4.5 cm there. In the corrugation zones (stations U1–U14), annual amplitudes remain at 7.5–8.0 cm, and semi-annual amplitudes are close to 1.0–1.5 cm. The 18.61-year amplitudes are minimally higher than in the case of seamounts (approx. 4.0–5.5 cm). The most diverse values were observed at points A7–A29, where annual amplitudes range from 7.0 to 9.5 cm, semi-annual amplitudes range from 0.3 to 2.5 cm, while 18.61-year amplitudes present the highest values in the entire study area, reaching from 10 to over 22 cm (e.g., A13—22.12 cm and A20—21.22 cm). This confirms the strong relationship between these long-term fluctuations and local tectonic and bathymetric conditions.

In our study, although the analysis period (1993–2021) covers only ~1.5 cycles of the 18.61-year cycle, an attempt was made to estimate it by fitting the appropriate harmonic component in the time series analysis of sea level. The amplitudes are significantly lower than for seasonal components (annual and semi-annual), which is consistent with expectations. However, their spatial variation offers insights into potential interactions between seafloor topography and the transmission of long-term tidal signals. In particular, points located on corrugations and transition zones (e.g., U- and A-type) presented slightly higher amplitudes of this component, which may indicate local effects or amplification of tidal influences by specific terrain features. In the future, the inclusion of the 18.61-year cycle in sea level change analyses has to be based on much longer measurement series, preferably exceeding 40–50 years. This will not only permit a more precise separation of this component but also support a systematic assessment of how its influence varies as a function of local bathymetric conditions, ongoing climate change, and the magnitude of extreme events. In this context, further research on the interactions between seafloor structure and long-term tidal modulation may prove crucial for improving regional sea level change forecasts and assessing the risk of coastal flooding.

**Figure 5:**
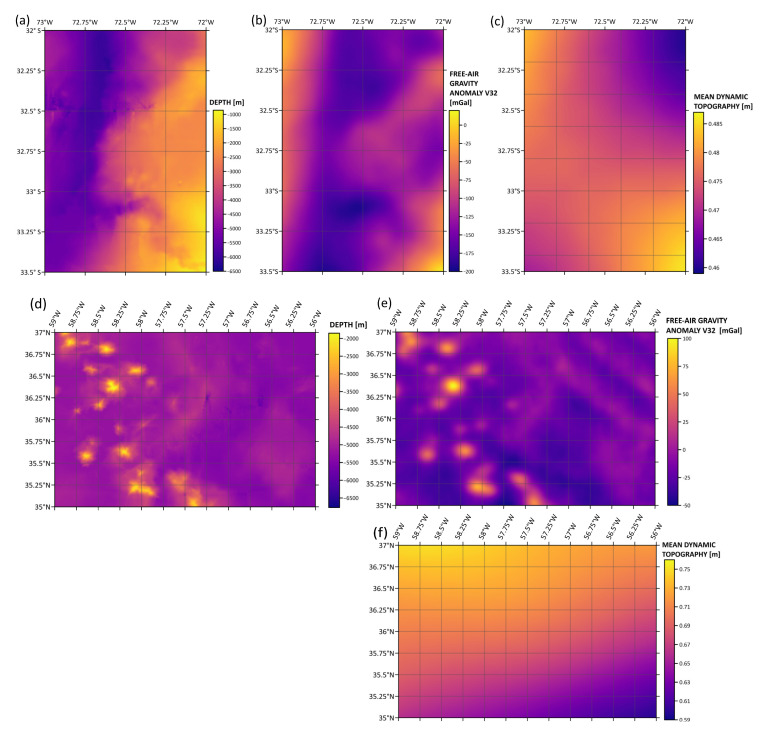
Seafloor and sea surface gridded models: depth model (**a**,**d**), free-air gravity anomaly V32 model (**b**,**e**), and mean dynamic topography (MDT) model (**c**,**f**).

The next step in the analysis of the relationship between the sea surface and seafloor structure was the generation of spatial grid models: depth, free-air gravity anomaly, and mean dynamic topography. The generated models are shown in Figure 5.

**Figure 6:**
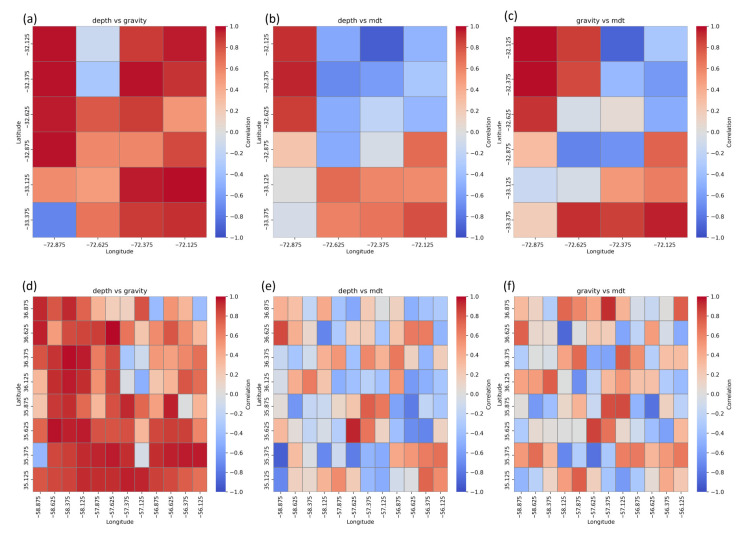
Correlograms—ocean trench and canyon region (**a**–**c**); seamounts and corrugation region (**d**–**f**).

In the Peru–Chile Trench and San Antonio Canyon region, the SIO V32 surface gravity anomaly model is overlaid with the depth models—in locations where depth is higher, marine gravity anomalies are less than 0 mGal. The MDT values are the highest in the submarine canyon location. In the second subregion, at the tops of the seamounts, gravity anomalies reach up to 100 mGal according to the SIO V32 gravity model. The highest values of mean dynamic topography (MDT) occur in the seamount locations. Interpolated surfaces were compared by determining the correlation coefficients, which were compiled in the form of correlograms. Spatial Pearson correlation maps are shown in Figure 6.

In the first subregion, covering the deep parts of the ocean trench (>4000 m), a moderate negative correlation was observed between the depth–gravity and depth–MDT model pairs. This may be evidence of the complex interaction of tectonics and seafloor topography on the gravity and on the dynamic conditions of the ocean surface. However, in the same area, a strong positive correlation between gravity anomalies and MDT was observed, confirming that free-air gravity anomalies are directly reflected in the dynamic field of the sea surface. In transition zones (1000–4000 m), characterized by varied seafloor relief, the depth–gravity relationship remains moderately positive, while the depth–MDT and gravity–MDT pairs present predominantly negative correlations, indicating a more complex interaction between seafloor structure and ocean circulation. In contrast, in shallow water areas (<1000 m) and within the submarine canyon, all three models show very strong positive correlations, suggesting a dominant role of direct seafloor topography in forming both the gravity and the dynamic surface of the ocean.

The second subregion, with lower depths and the presence of bathymetric elevations (seamounts) and corrugations, is characterized by more diverse relationships. In the deepest parts of this area, the depth–gravity relation remains positive but weak, while the depth–MDT and gravity–MDT pairs reach moderate values with variable polarity (positive or negative), indicating a higher role of local tectonic and hydrodynamic factors. In transition zones, where there are significant changes in depth, very high positive correlations between depth and gravity anomalies are observed. The rest model pairs maintain moderate or low values. In turn, in shallow areas, especially at the tops of seamounts, a highly strong positive correlation was observed in depth–gravity pairs, with very low and variable (positive or negative) correlations between MDT and gravity anomalies. This combination suggests that in areas with bathymetric elevations, it is mainly the seafloor topography that determines the distribution of the gravity field, while the influence on the dynamic conditions of the sea surface remains limited and more heterogeneous.

In summary, in deeper parts of the ocean trench (T), low positive correlations are a result of high tectonic complexity. In areas of seamounts and corrugations (S and U), the strong depth–gravity correlation with simultaneously low values in pairs with MDT reflects differences in the scale and nature of the models—MDT smooths out small features of the seafloor relief. In canyons (C), tectonic and erosive processes dominate, resulting in a strong positive correlation in all models. The inclusion of densification points (A) confirms that the observed patterns are not random but result from the bathymetric and geological specificity of the area. These differences in correlations indicate that the accuracy of oceanographic models and surface circulation forecasts may be strongly dependent on the local seabed structure, which highlights the need to take bathymetry diversity into account in modeling and interpreting MDT data.

## 6. Discussion

Dynamic and thermodynamic changes in the ocean are crucial in regional sea level changes. Cazenave et al. [72] state that at the regional scale, ocean thermal expansion is still the main cause of the spatial trend patterns observed by satellite altimetry. Mean sea level rise is mainly due to heat absorption by the oceans, which leads to thermal expansion and an increase in the total mass of the ocean. Mean sea level rise is also an important feature of anthropogenic climate change due to atmospheric and ocean circulation [73,74,75]. According to Conrad [76], sea level fluctuations are global and related to seafloor topography. Accordingly, this paper assesses the variability of the sea surface regarding the forms of the seafloor (ocean trench, canyon, seamounts chain, and corrugations) and models the correlation between depth, free-air gravity anomalies, and mean dynamic topography (MDT). This study used hybrid datasets: satellite altimetry time series, bathymetric echosoundings, GEBCO model, marine gravity anomaly model (SIO V32), and DTU 2022 Mean Dynamic Topography model.

**Figure 7:**
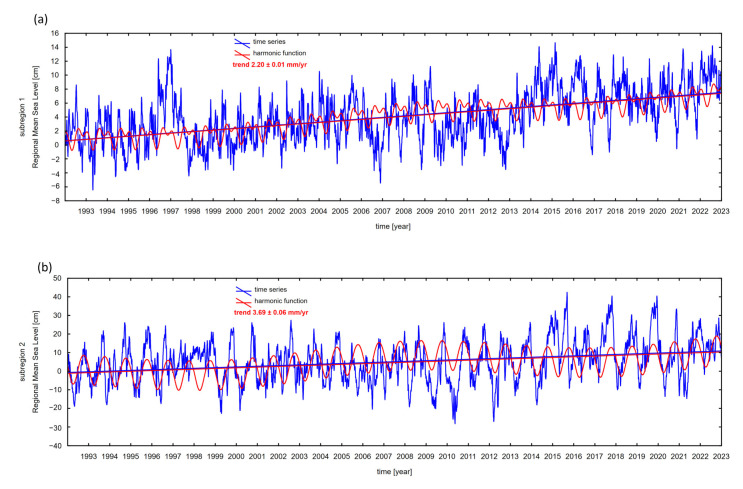
Time series of mean sea level anomalies based on satellite altimetry data for subregion 1 (**a**), and subregion 2 (**b**).

**Figure 8:**
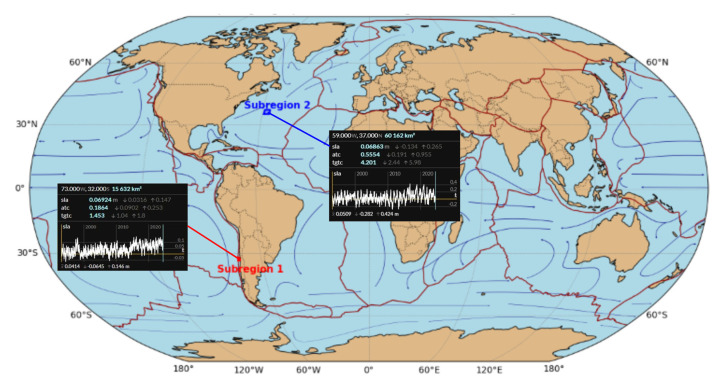
Sea level change trends and acceleration generated by ocean data visualization tools.

Differences in trends of sea level changes are spatially and temporally variable. The regional mean sea level trend in the trench and submarine canyon region is equal to 2.20 ± 0.01 mm/year. In this region, the trend values of individual virtual observation points are stable and spatially similar. In the seamounts and corrugation region, trend values are more irregular than in the trench and canyon area. The regional mean sea level trend in the seamounts and corrugation region is 3.69 ± 0.06 mm/year (Figure 7). Mean sea level trends have been compared with trends and acceleration, generated by Copernicus ocean data visualization tools (https://marine.copernicus.eu/access-data/ocean-visualisation-tools—accessed on 16 April 2025) based on the OMI_CLIMATE_SL_GLOBAL_regional_trends product (Figure 8). Trend values display a high degree of similarity.

The observed rates of sea level change directly result from the location within the tectonic plate zone and the ocean currents in the regions of Peru–Chile Current (subregion 1) and the Gulf Stream Current and North Atlantic Drift (subregion 2). The cold Peru–Chile Current transports colder waters, leading to lower changes in sea level. In contrast, the warm Gulf Stream and North Atlantic Drift tend to expand ocean waters, leading to higher sea level changes. Sea level dynamics are regulated by oceanographic, atmospheric, hydrological, and geological processes; therefore, the values in sea level trends are different in both subregions.

The results of the harmonic analysis indicate that sea level variability in the studied areas is closely related to the local seafloor topography. Lower annual and semi-annual amplitudes occurring in ocean trenches and canyons indicate that short-term fluctuations are dampened by their complex topography. In contrast, higher amplitudes are observed in areas of corrugation and seamounts, suggesting an amplification of cyclical fluctuations in response to local bathymetric conditions. The variations confirm the importance of incorporating seafloor topography and oceanographic processes into the analysis of sea level changes.

Our results have important implications for oceanographic models and climate predictions. Correlation analysis between depth, gravity anomalies, and mean dynamic topography (MDT) in different subregions emphasizes that local seafloor topography significantly influences gravity fields and dynamic conditions at the sea surface. Observed correlation coefficients—including a strong positive correlation between gravity and MDT in deep ocean areas—were further subjected to critical analysis. The results suggest that although this relation is significant, it may also be modulated by isostatic mass equalization and local differences in the density distribution of the oceanic crust [77].

Throughout the analyzed period, the anomaly jumps are associated with the well-documented 1997–1998 El Niño phenomena, which markedly contributed to regional sea level rise in the eastern Pacific by halting upwelling and accumulating warm water along the coast of South America [78,79]. Studies present that the sea level response to ENSO in this region can reach ±20 cm relative to neutral conditions [80]. ENSO affects sea level through both thermal (thermal expansion) and dynamic mechanisms, modifying atmospheric pressure and wind distribution, which causes the displacement of water masses along the Equator. These mechanisms are consistent with the observed high amplitude of anomalies in subregion 1, confirming that long-term regional trends are modulated by short-period but intense climate events [81,82,83].

The variability of amplitudes in annual, semi-annual, and 18.61-year cycles indicates a significant influence of the seafloor topography. In seamount areas, amplitudes reach their highest values, which can be interpreted as the effect of cyclical fluctuations amplified by bathymetric elevations. Lower amplitudes are observed in ocean trenches and canyons, suggesting that oscillations are dampened by the complex relief and sedimentary structure of the seafloor. In corrugation zones, amplitudes take on intermediate values, which emphasize local modulations of tidal processes. Such variation indicates that amplitude analysis can be a valuable source of information about the interaction between oceanographic processes and seafloor topography [84].

An increasing number of studies demonstrate that bathymetry affects local variations in sea level anomalies (SLAs), modifying the propagation of global climate change signals [85,86]. Studies have shown that bathymetric structures can dampen or modify the propagation of sea level anomaly (SLA) signals. Incorporating detailed bathymetry into ocean models improves the agreement between simulation results and both satellite data and in situ measurements, thereby increasing the reliability and accuracy of sea level analyses [87,88].

Within the framework of dynamic and thermodynamic process interactions, it is important to note that regional sea level trends highly depend on a combination of circulation processes. Changes in wind strength, evaporation and precipitation balance, freshwater flows, and heat absorption by the ocean affect the distribution of mass and heat in the ocean, leading to spatial changes in SLA. Models such as ENSO, IOD (Indian Ocean Dipole), and NAO (North Atlantic Oscillation) further modulate these processes on an interannual and decadal scale [89,90,91,92].

**Figure 9:**
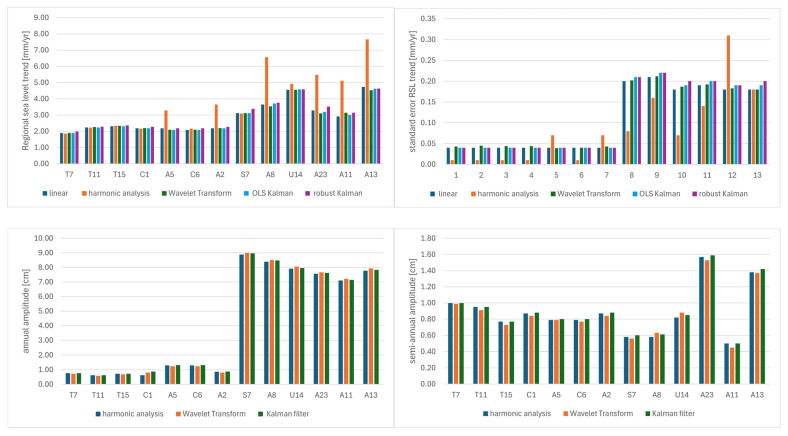
Results validation.

Recent studies also emphasize that the vertical structure of the ocean is important for the response of regional sea level to dynamic and thermodynamic forcings. In deep areas, such as the Peru–Chile Trench (subregion 1), changes in steric height occur more slowly and are modulated by heat absorption in the deep ocean and internal mixing. By contrast, in shallow or topographically complex areas—such as subregion 2—responses tend to be quicker and more variable [93,94]. Moreover, human activities—such as coastal development, river damming, and groundwater extraction—can indirectly influence sea level by causing land subsidence and altering the water cycle. Therefore, a comprehensive approach to analysis is essential, integrating dynamic (e.g., ocean circulation) and thermodynamic (e.g., thermal expansion) components [95,96,97]. The results of the validation (Figure 9) of the harmonic analysis (at a few selected points) using the wavelet transform and Kalman filtering methods indicate a high degree of consistency between the methods, especially in terms of linear trends.

The highest consistency of trends was obtained at points T7, T11, and T15, where the differences between the OLS, Fourier, and Kalman trend values were minimal (e.g., T11: OLS trend = 2.24 mm/year; Kalman = 2.29 mm/year). Annual and semi-annual amplitudes also present high repeatability, although their values were strongly modulated by local bathymetric conditions. In subregion 2 (e.g., A8, U14), significantly higher annual amplitudes (7.92–8.48 cm) are observed with low divergence between methods (differences < 0.1 cm). The WT method also allowed for the estimation of acceleration—e.g., at point A8, the acceleration reached a value of 0.40 mm/year^2^, which may indicate non-linear changes in sea level. Applying Kalman filters (both OLS and robust) enhanced the detection of local fluctuations and potential seasonal effects, while also increasing resistance to data noise—an important factor for forecasting and modeling coastal trends. The strong agreement among the results from the three independent methods underscores the robustness of the estimates and supports the reliability of the conclusions.

Finally, the study results have practical applications, such as in coastal zone management, adaptation to rising sea levels, and the development of regional climate change mitigation strategies. At the same time, it is important to acknowledge methodological limitations—for example, the impact of altimetry data quality on trend accuracy, possible errors in MDT estimation, or spatial resolution heterogeneity of data. These limitations ought to be acknowledged in the interpretation of results and the design of future studies.

## 7. Conclusions

Regional sea level variability presents significant spatial differences between two subregions: subregion 1—the west coast of South America (Peru–Chile Trench and the San Antonio submarine canyon), subregion 2—the seamount ranges and corrugations (New England Seamounts and Corner Rise Seamount Complex).

In the study area, sea level changes present spatial variation, highly correlated with the local seafloor topography. In studies of the relationship between seafloor topography and sea level change, gravity anomalies have been used as an indirect source of information about seafloor topography. These anomalies reflect changes in the distribution of mass, which affects the shape of the gravitational field and can cause local differences in sea level due to geostatic and dynamic effects. Integrating the analysis of gravitational anomalies with bathymetric data and sea level observations enables more comprehensive modeling of ocean processes.

Ocean trench and canyon areas are characterized by lower seasonal amplitudes and more stable sea level trends, while regions of seamounts and corrugation show higher dynamics, higher amplitudes, and faster sea level rise.

The regional sea level trend in seamounts and corrugation zones reached up to 7.66 mm/year, more than twice as high as in deep locations (1.75–3.65 mm/year). This variation reflects the influence of local seafloor topography on regional ocean mass and heat balances.

The harmonic analysis carried out in this study confirmed the presence of seasonal cycles (annual and semi-annual), whose amplitudes depend on the type of seafloor topography. The highest values were recorded in areas of seamounts, indicating possible interactions between bathymetry and the circulation and structure of water layers.

Fluctuations in the 18.61-year cycle present spatial differences—the highest amplitudes (more than 22 cm) were observed in transition zones and above seamounts, which may indicate local amplification of the long-term tidal signal.

Correlogram analysis suggests that the relationships between depth, gravity anomalies, and MDT vary significantly depending on the seafloor topography. Low or moderate correlations were observed in deep parts of the trench, which may be the result of complex tectonics, while on seafloor elevations, depth–gravity relationships were strong, with weaker relationships with MDT.

The use of harmonic analysis, Wavelet Transformation, and Kalman filter methods increased the reliability of the results and allowed for a more complex understanding of the regional sea level variability.

The influence of climatic phenomena such as ENSO was clearly noted in temporal anomalies, e.g., in the period 1997–1998. This indicates that regional sea level changes are modulated not only by seafloor topography but also by dynamic and thermal factors with variable time scales.

The main objective of this study was to determine how local seafloor topography affects regional sea level trends and amplitudes. The results confirm that seafloor topography is one of the key factors forming ocean surface dynamics, which is important for future oceanographic models and forecasting the effects of climate change.

## Data Availability

Satellite altimetry data were provided by the Copernicus Marine Environment Monitoring Service (https://data.marine.copernicus.eu/products)—accessed on 20 May 2024. Multibeam bathymetry data were provided by the NOAA National Centers for Environmental Information (NCEI) (https://www.ncei.noaa.gov/maps/iho_dcdb/)—accessed on 15 February 2023. Global, digital elevation GEBCO_22 model was provided by the General Bathymetric Chart of the Oceans (https://www.gebco.net/data_and_products/gridded_bathymetry_data/)—accessed on 6 March 2023. Free-air Gravity Anomaly Model V32 was provided by the Scripps Institution of Oceanography (SIO) (https://topex.ucsd.edu/pub/)—accessed on 19 July 2024. Mean Dynamic Topography Model—DTU22MDT model was provided by the Technical University of Denmark (DTU) (https://data.dtu.dk/)—accessed on 5 April 2025.

## Abbreviations

The following abbreviations are used in this manuscript:

CMEMS	Copernicus Marine Environment Monitoring Service
GEBCO	General Bathymetric Chart of the Oceans
SIO	Scripps Institution of Oceanography
NOAA NCEI	National Oceanic and Atmospheric Administration, National Centers for Environmental Information
SLA	Sea Level Anomaly
MDT	Mean Dynamic Ocean Topography
